# Health-Related Social Needs and Health Care Utilization in the Accountable Health Communities Model

**DOI:** 10.1001/jamanetworkopen.2025.48036

**Published:** 2025-12-15

**Authors:** Abbey C. Sidebottom, Summer Martins, Marc C. Vacquier, Christy Dechaine, Dan Behrens

**Affiliations:** 1 Care Delivery Research, Allina Health, Minneapolis, Minnesota; 2 Clinical Research Informatics and Analytics, Allina Health, Minneapolis, Minnesota; 3 Community Benefit and Engagement, Allina Health, Minneapolis, Minnesota; 4 Primary Care Administration, Allina Health, Minneapolis, Minnesota

## Abstract

**Importance:**

A better understanding of how health-related social needs (HRSNs) are associated with health care utilization may help health systems prioritize efforts to address HRSNs.

**Objectives:**

To examine the association of HRSNs with inpatient (IP) and emergency department (ED) utilization and whether resolution of HRSNs is associated with a reduction in utilization.

**Design, Setting, and Participants:**

This retrospective cohort study included 166 682 Medicare and Medicaid recipients in a single health system who were screened for HRSNs under the Accountable Health Communities (AHC) program from June 5, 2018, to January 31, 2022. A follow-up sample included the subset of patients with multiple screenings in the study period. Statistical analysis was performed from June 2024 to September 2025.

**Exposures:**

Health-related social needs assessed under the AHC program included housing stability, housing quality, food security, transportation, utilities, and interpersonal safety. Measures included the presence of individual HRSNs, number of needs, and resolution of need at follow-up.

**Main Outcomes and Measures:**

IP admissions and ED visits were ascertained for 6 months before baseline screening and 6 months after follow-up. Models assessed the association of baseline HRSNs and IP and ED admissions in the 6 months prior to screening, and resolution of HRSNs with IP and ED admissions in the 6 months after follow-up. Models were mutually adjusted for HRSNs as well as demographic characteristics and comorbidities.

**Results:**

There were 166 682 patients (43.4% aged ≥65 years; 60.3% women) with baseline screening in the sample. All 6 baseline HRSNs were associated with ED use after adjustment for all covariates: 5 with higher odds of ED visits and 1 (housing quality) with lower odds of ED use; point estimates were largest for housing stability (odds ratio [OR], 1.25; 95% CI, 1.18-1.33) and transportation (OR, 1.31; 95% CI, 1.25-1.38). Emergency department visits occurred for 26.9% of patients with housing stability needs and 25.6% of those with transportation needs. After adjustment for other HRSNs, demographic characteristics, and health conditions, only 2 HRSNs retained an association with an increase in IP admissions: housing stability (OR, 1.34; 95% CI, 1.23-1.47) and transportation (OR, 1.16; 95% CI, 1.08-1.24). In the subsample with follow-up screening (n = 15 139), resolution of HRSNs was not associated with utilization after adjustment for other HRSNs, demographic characteristics, and comorbidities.

**Conclusions and Relevance:**

In this cohort study, most HRSNs were associated with ED visits, with strongest associations for housing instability and transportation. Associations were not present for adjusted models for resolution of HRSNs. Further research on the fluctuation of individual HRSNs over time is warranted to better understand if resolution of needs is associated with reduced health care utilization.

## Key Points

**Question:**

Are individual health-related social needs (HRSNs) associated with inpatient and emergency department (ED) visits?

**Findings:**

In this cohort study of 166 682 Medicaid and Medicare recipients, 5 of 6 HRSNs were associated with higher ED use at baseline, and 2 HRSNs (housing stability and transportation) were associated with inpatient admissions after adjusting for confounders. Resolution of HRSNs was not associated with health care utilization after adjusting for other HRSNs, demographic characteristics, and comorbidities.

**Meaning:**

This study suggests that addressing specific HRSNs may help reduce the need for acute health care utilization.

## Teaser

This cohort study of Medicare and Medicaid recipients examines the association of health-related social needs with inpatient and emergency department utilization and whether resolution of health-related social needs is associated with a reduction in utilization.

## Introduction

Health-related social needs (HRSNs) are unmet social factors that may affect health outcomes, health care costs, health disparities, and quality of life.^1,2,3,4^ Health-related social needs center on basic needs such as food, housing, and interpersonal safety.^5^ Health-related social needs are the individual-level manifestation of societal, environmental, economic factors often defined as social determinants of health. Although social determinants of health are structural factors often measured at the community level and explain health inequalities between populations, HRSNs are social and economic needs experienced at the individual level.^1,6,7^ Socioeconomic circumstances may explain up to 40% of health outcomes independently of behaviors, genetics, and medical care.^1,8^

The rationale for addressing HRSNs in health care settings comes from multiple perspectives. First, there is an expanded understanding of the role of care systems in supporting patients’ health holistically, addressing factors outside of the clinical context that affect health outcomes. In addition, a market-based perspective such as the movement toward value-based reimbursement models incentivizes health systems to implement initiatives focused on reducing health care costs and improving quality of care and support establishing additional reimbursement models for community-based organizations providing social services.^3^ Given the strong association between HRSNs and health status, health care systems are moving to better identify and address HRSNs.^3^ In support of these efforts, the Centers for Disease Control and Prevention developed diagnostic codes specific to social determinants of health and expanded guidelines to encourage use of these codes.^9^ However, assessing patients’ HRSNs and connecting them with related resources is not yet part of standard clinical care.^5^

Several individual HRSNs have demonstrated associations with health care utilization: housing instability with an increase in emergency department (ED) visits and postponed medications; food insecurity with missed well-child visits and an increase in ED visits; and physical abuse with an increase in utilization of ED, primary care, and specialty care visits.^10,11,12,13^ Some interventions have tested if addressing a specific HRSN such as housing or food insecurity may improve health status and reduce ED or inpatient (IP) visits.^14,15,16,17^ A Chicago-based randomized clinical trial tested transitional housing services provided at hospital discharge for unhoused patients, followed by long-term housing, and found substantially reduced health care utilization compared with standard discharge planning.^16^ Among this population of unhoused patients, those randomized to housing services experienced rates of hospitalizations 29% lower, and rates of ED use 24% lower, relative to the comparison population over 18 months. A meal delivery program for vulnerable adults (dually eligible for Medicaid and Medicare), found reduced ED and IP utilization and health care expenditures for those receiving both medically tailored meals and nontailored meals compared with nonparticipants.^15^ A review of programs addressing HRSNs identified multiple interventions with similar findings when addressing specific needs of housing, food, or transportation, as well as multidisciplinary care management programs.^17^

The Accountable Health Communities (AHC) model was developed by the Centers for Medicare & Medicaid Services (CMS) to systematically assess Medicaid and Medicare recipients for HRSNs and establish pathways to addressing those needs through partnerships between AHC sites and local community organizations.^5,18^ The comprehensive screening process implemented in the AHC model enables the examination of the association of multiple HRSNs with health care utilization and health status within defined geographic and health system populations. To date, research examining HRSNs in the AHC model (or AHC screening tool) has identified an association of co-occurring HRSNs with an increase in ED utilization^19^ and an association of specific HRSNs with higher care utilization for patients with type 2 diabetes^13^ and older adults.^20^ Studies examining the association of particular HRSNs with care-seeking behavior in the general adult population are lacking. In addition, no studies have examined whether resolution of HRSNs previously documented with the AHC screening tool results in lower utilization of health care services.

This study uses data from 1 AHC program to examine the association between HRSNs and health care utilization in the context of a large health system. The goals of the study were to (1) examine how HRSNs may be associated with IP admissions and ED visits and (2) examine if resolution of HRSNs may be associated with lower utilization of IP or ED services. By addressing these questions, we hope to aid health systems with the development of models to address HRSNs and to prioritize risk stratification based on specific needs and how they are associated with care utilization.

## Methods

### Setting

The AHC model was a 5-year pilot cohort study funded by CMS testing whether “systematically identifying and addressing health-related social needs can reduce health care costs and utilization among community-dwelling Medicare and Medicaid beneficiaries.”^21^ From June 5, 2018, through January 31, 2022, Allina Health screened patients for HRSNs under this model. Allina Health is a nonprofit health system serving Minnesota and western Wisconsin. Screening was offered to all patients insured by Medicaid or Medicare at 79 primary care, urgent care, obstetrics-gynecology, and mental health clinics as well as select departments of 3 metropolitan hospitals (selected for higher Medicaid and Medicare patient populations). Screening was conducted with the AHC screening questionnaire^5^ completed on paper by patients. Front desk staff in clinics and nursing or otherwise designated staff within the hospitals identified patients eligible for the screening and asked them to complete the questionnaire, then entered the results into the electronic health record (EHR). Starting in 2020, to address the substantial increase in virtual visits due to the COVID-19 pandemic, MyChart screenings (with a link to questionnaire) and telephonic screenings were also offered. The use of these data for research was determined to be exempt by the Allina Health institutional review board, and a waiver of informed consent was granted. We followed the Strengthening the Reporting of Observational Studies in Epidemiology (STROBE) reporting guideline.^22^

If a patient identified HRSNs, the care team provided a community referral summary—an automatically generated list of community resources tailored to the patient’s needs and geographic area produced through a web-based software called NowPow.^23^ Under the AHC model, patients who screened positive for HRSNs and self-reported 2 or more ED visits in the last year were identified as high risk. A subset of patients identified as high risk was randomized to receive optional navigation services to connect with community resources to address their needs.^24^ Patients could be screened as often as every 6 months as aligned with appointments. Outcomes of subsequent screenings could change patients’ eligibility for navigation services, but once a patient was randomized to control or intervention groups, they remained in that group for the duration of the study. Over the course of the program, the proportion of eligible patients or visits who were offered screening by staff and the proportion completing a screening were tracked and incorporated into staff training and quality improvement to increase in screening offer rates over time.

### Study Design and Sample

Patients were included in the study if they completed at least 1 HRSN screening between June 5, 2018, and January 31, 2022, regardless of whether they were randomized to optional navigation services. To address the first study aim, we conducted a cross-sectional analysis to examine the association of HRSNs with IP and ED visits at baseline for the full sample. To address the second study aim, we narrowed the sample to the subset of patients who had at least 2 screenings so that we could assess whether HRSNs were resolved between baseline and follow-up screening.

### Data Collection and Measures

The AHC screening tool assessed HRSNs in 5 domains: housing (instability, problems with quality), food (worry about running out, no money to get more), transportation (unreliable), utilities (company threatened to shut off service), and interpersonal safety (physical or verbal abuse from anyone).^5^ Using instrument scoring instructions, we classified participants as having or not having a need in each domain except for housing; we classified housing stability and quality (eg, pests, mold, leaks) separately for a total of 6 HRSNs. Questions referred to the last 12 months (food, transportation, and utilities) or current status (housing and interpersonal safety). The presence or absence of each need was classified at baseline screening and the earliest follow-up screening 6 months or more after baseline. Needs were considered resolved if present at baseline but absent at follow-up.

Additional measures were pulled from the EHR. Health care utilization—IP admissions and ED visits—was extracted for 6 months before and after each HRSN screening. Patient characteristics at baseline screening included sex, race and ethnicity, age, insurance payer, preferred language, and health conditions. Race and ethnicity were self-reported verbally as part of the patient registration process in the clinical setting. Race and ethnicity were collected as part of the AHC model cooperative agreement and for internal analysis purposes to understand distribution of HRSNs by race and ethnicity. Patients could report more than 1 race. Race and ethnicity were combined to categorize patients into non-Hispanic American Indian or Alaska Native (hereinafter American Indian or Alaska Native), non-Hispanic Asian or Pacific Islander (hereinafter Asian or Pacific Islander), non-Hispanic Black (hereinafter Black), Hispanic or Latino (any race), non-Hispanic White (hereinafter White), non-Hispanic multiracial (hereinafter multiracial), and declined to answer or missing. Payers were classified as Medicaid, Medicare, dual eligible, or other. The latter category includes patients who were previously covered by Medicaid or Medicare (and thus eligible for AHC screening) but used private insurance for the screening encounter. Language comprised the 3 most common languages (English, Somali, and Spanish), with the remaining grouped as other. Health conditions were identified using *International Statistical Classification of Diseases and Related Health Problems, Tenth Revision* (*ICD-10*), diagnosis codes and the EHR problem list. We included conditions that were hypothesized to be associated with the likelihood of having HRSNs and/or utilizing care: asthma, type 2 diabetes, depression, hypertension, chronic kidney disease, chronic liver disease, chronic obstructive pulmonary disease, substance abuse, alcohol abuse, or cardiovascular disease.^25,26,27,28,29^

### Statistical Analysis

Statistical analysis was performed from June 2024 to September 2025. Patient characteristics were summarized with descriptive statistics. For all characteristics, we computed the prevalence of having any HRSN (composite) as well as each of the HRSNs within each variable level (eg, female, Hispanic) and assessed significant differences using 1-sided χ^2^ tests; *P* ≤ .05 was considered significant. Using a similar approach, we examined bivariate associations between HRSNs and having an IP admission or ED visit in the 6 months prior to the baseline screening. We used retrospective measures of utilization for the baseline analyses to align them with the reference windows used for most HRSN measures.

Two sets of multivariable logistic regression models were conducted to examine the independent association between HRSNs and health care utilization. First, we assessed cross-sectional associations at baseline by estimating adjusted odds ratios (ORs) for having an IP admission or ED visit in the 6 months before baseline screening with baseline HRSN as the exposure. We estimated 3 models for each outcome: model 1 was mutually adjusted for all of the 6 HRSNs at baseline, model 2 added covariates for demographic characteristics, and model 3 added covariates for comorbidities. This nested approach allowed us to assess the relative contribution of each category; final models retained all covariates.

Second, we evaluated whether resolution of HRSNs identified at baseline was associated with subsequent changes in health care utilization. For these follow-up analyses, samples were restricted to patients who identified the HRSN at baseline and completed at least 2 screenings to classify baseline HRSNs as being resolved or not at follow-up screening. Odds ratios for IP admissions and ED visits were estimated separately for the 6-month window after follow-up screening. Initial models were adjusted for the presence of other HRSNs at follow-up screening. Similar to the baseline analysis, models were subsequently adjusted for demographic covariates (model 2) and then comorbidities (model 3). Analyses were performed in Stata, version 18.0 (Stata Corp LLC).

## Results

A total of 166 682 unique patients (43.4% aged ≥65 years; 60.3% women and 39.7% men; 0.6% American Indian or Alaska Native, 3.9% Asian or Pacific Islander, 12.0% Black, 5.6% Hispanic or Latino, 73.0% White, 1.7% multiracial, and 3.3% declined to answer or missing) were screened for HRSNs during the study period (Table 1). During the study period, there were 378 413 unique eligible patients who had at least 1 visit at a study clinic or hospital; of those, 299 032 (79.0%) were offered screening at a visit, and 166 682 (55.7%) of those completed a screening. Of the 166 682 patients completing screening, 40 504 (24.3%) had 1 or more need identified at baseline (Table 1). Prevalence of needs ranged from 1.4% (n = 2365; interpersonal safety) to 14.5% (n = 24 106; food). Prevalence varied significantly across demographic variables for the composite measure as well as all 6 individual HRSNs. Subgroups with a particularly high prevalence of HRSNs were multiracial or American Indian patients; patients aged 18 to 44 years or 45 to 64 years; those dually insured by Medicaid and Medicare; and Spanish speakers. Health-related social needs were significantly more common among patients with asthma, depression, chronic liver disease, alcohol abuse, and substance abuse (Table 2).

**Table 1.** Patient Characteristics

Characteristic	Patients, total No. (%)^a^	HRSN, No. (%)^b^						
		Any need	Housing stability	Housing quality	Food	Transportation	Utilities	Interpersonal safety
Total	166 682	40 504 (24.3)	7463 (4.5)	13 077 (7.9)	24 106 (14.5)	13 716 (8.2)	9104 (5.5)	2365 (1.4)
Sex								
Female	100 562 (60.3)	26 199 (26.1)^c^	4662 (4.6)^c^	8485 (8.4)^c^	15 821 (15.7)^c^	8935 (8.9)^c^	5952 (5.9)^c^	1673 (1.7)^c^
Male	66 120 (39.7)	14 305 (21.6)	2801 (4.2)	4592 (7.0)	8285 (12.5)	4781 (7.2)	3152 (4.8)	692 (1.1)
Race and ethnicity								
American Indian or Alaska Native	996 (0.6)	425 (42.7)^c^	119 (12.0)^c^	126 (12.7)^c^	287 (28.8)^c^	216 (21.7)^c^	116 (11.7)^c^	36 (3.6)^c^
Asian or Pacific Islander	6449 (3.9)	1622 (25.2)	263 (4.1)	522 (8.1)	1012 (15.7)	409 (6.3)	222 (3.4)	62 (1.0)
Black	19 948 (12.0)	6975 (35.0)	1298 (6.5)	1722 (8.6)	4580 (23.0)	2811 (14.1)	1746 (8.8)	401 (2.0)
Hispanic or Latino	9292 (5.6)	3229 (34.8)	524 (5.6)	941 (10.1)	2048 (22.0)	1094 (11.8)	749 (8.1)	136 (1.5)
White	121 609 (73.0)	25 568 (21.0)	4762 (3.9)	8898 (7.3)	14 505 (11.9)	8254 (6.8)	5565 (4.6)	1587 (1.3)
Multiracial	2860 (1.7)	1118 (39.1)	218 (7.6)	376 (13.2)	733 (25.6)	428 (15.0)	295 (10.3)	67 (2.3)
Declined to answer or missing	5528 (3.3)	1567 (28.4)	279 (5.1)	492 (8.9)	941 (17.0)	504 (9.1)	411 (7.4)	76 (1.4)
Age, y								
≤17	31 401 (18.8)	8565 (27.3)^c^	1122 (3.6)^b^^c^	2530 (8.1)^c^	5120 (16.3)^c^	2417 (7.7)^c^	2499 (8.0)^c^	308 (1.0)^c^
18-44	40 675 (24.4)	15 954 (39.2)	3514 (8.6)	4822 (11.9)	10 483 (25.8)	6003 (14.8)	3710 (9.1)	1198 (3.0)
45-64	22 207 (13.3)	8794 (39.6)	2060 (9.3)	2708 (12.2)	5889 (26.5)	3256 (14.7)	2011 (9.1)	645 (2.9)
65-84	65 207 (39.1)	6675 (10.2)	725 (1.1)	2835 (4.4)	2485 (3.8)	1841 (2.8)	839 (1.3)	204 (0.3)
≥85	7192 (4.3)	516 (7.2)	42 (0.6)	182 (2.5)	129 (1.8)	199 (2.8)	45 (0.6)	10 (0.1)
Payer								
Dual	6241 (3.7)	2562 (41.1)^c^	489 (7.8)^c^	740 (11.9)^c^	1828 (29.3)^c^	1105 (17.7)^c^	470 (7.5)^c^	200 (3.2)^c^
Medicaid	83 663 (50.2)	28 953 (34.6)	5852 (7.0)	8684 (10.4)	18 452 (22.1)	9915 (11.9)	7369 (8.8)	1783 (2.1)
Medicare	73 139 (43.9)	7911 (10.8)	975 (1.3)	3310 (4.5)	3157 (4.3)	2310 (3.2)	1115 (1.5)	329 (0.5)
Other^d^	3639 (2.2)	1078 (29.6)	147 (4.0)	343 (9.4)	669 (18.4)	386 (10.6)	150 (4.1)	53 (1.5)
Language								
English	157 426 (94.4)	38 338 (24.4)^c^	7127 (4.5)^c^	12 605 (8.0)^c^	22 907 (14.6)^c^	12 910 (8.2)^c^	8775 (5.6)^c^	2327 (1.5)^c^
Somali	2644 (1.6)	396 (15.0)	35 (1.3)	74 (2.8)	143 (5.4)	184 (7.0)	66 (2.5)	8 (0.3)
Spanish	2265 (1.4)	762 (33.6)	127 (5.6)	180 (8.0)	474 (20.9)	239 (10.6)	145 (6.4)	9 (0.4)
Other	4347 (2.6)	1008 (23.2)	174 (4.0)	218 (5.0)	582 (13.4)	383 (8.8)	118 (2.7)	21 (0.5)

Abbreviation: HRSN, health-related social need.

^a^ Percentages are column percentages.

^b^ Percentages of individual HRSNs are row percentages derived from denominators in the patient total column.

^c^ *P* < .001.

^d^ Includes any insurance other than Medicaid or Medicare.

**Table 2.** Prevalence of Health-Related Social Needs (HRSNs) at Baseline by Patient Health Condition

Health condition	Patients, total No.^a^	HRSN, No. (%) (N = 166 682)						
		Any need	Housing stability	Housing quality	Food	Transportation	Utilities	Interpersonal safety
Asthma								
Yes	19 428	6750 (34.7)^b^	1354 (7.0)^b^	2267 (11.7)^b^	4420 (22.8)^b^	2575 (13.3)^b^	1638 (8.4)^b^	538 (2.8)^b^
No	147 254	33 754 (22.9)	6109 (4.2)	10 810 (7.3)	19 686 (13.4)	11 141 (7.6)	7466 (5.1)	1827 (1.2)
Type 2 diabetes								
Yes	22 524	5237 (23.3)^b^	937 (4.2)^c^	1685 (7.5)^d^	3060 (13.6)^b^	1897 (8.4)	1072 (4.8)^b^	279 (1.2)^e^
No	144 158	35 267 (24.5)	6526 (4.5)	11 392 (7.9)	21 046 (14.6)	11 819 (8.2)	8032 (5.6)	2086 (1.5)
Depression								
Yes	43 021	15 351 (35.7)^b^	3444 (8.0)^b^	5020 (11.7)^b^	9942 (23.1)^b^	5836 (13.6)^b^	3454 (8.0)^b^	1307 (3.0)^b^
No	123 661	25 153 (20.3)	4019 (3.3)	8057 (6.5)	14 164 (11.5)	7880 (6.4)	5650 (4.6)	1058 (0.9)
Hypertension								
Yes	59 815	11 022 (18.4)^b^	2024 (3.4)^b^	3776 (6.3)^b^	6087 (10.2)^b^	3898 (6.5)^b^	2213 (3.7)^b^	570 (1.0)^b^
No	106 867	29 482 (27.6)	5439 (5.1)	9301 (8.7)	18 019 (16.9)	9818 (9.2)	6891 (6.5)	1795 (1.7)
Chronic kidney disease								
Yes	10 821	1844 (17.0)^b^	283 (2.6)^b^	602 (5.6)^b^	954 (8.8)^b^	651 (6.0)^b^	281 (2.6)^b^	75 (0.7)^b^
No	155 861	38 660 (24.8)	7180 (4.6)	12 475 (8.0)	23 152 (14.9)	13 065 (8.4)	8823 (5.7)	2290 (1.5)
Chronic liver disease								
Yes	4714	1436 (30.5)^b^	327 (6.9)^b^	455 (9.7)^b^	947 (20.1)^b^	577 (12.2)^b^	331 (7.0)^b^	110 (2.3)^b^
No	161 968	39 068 (24.1)	7136 (4.4)	12 622 (7.8)	23 159 (14.3)^b^	13 139 (8.1)	8773 (5.4)	2255 (1.4)
Chronic obstructive pulmonary disease								
Yes	9734	2543 (26.1)^b^	409 (4.2)	873 (9.0)^b^	1555 (16.0)^b^	949 (9.8)^b^	439 (4.5)^b^	154 (1.6)
No	156 948	37 961 (24.2)	7054 (4.5)	12 204 (7.8)	22 551 (14.4)	12 767 (8.1)	8665 (5.5)	2211 (1.4)
Cardiovascular disease								
Yes	72 387	13 605 (18.8)^b^	2518 (3.5)^b^	4702 (6.5)^b^	7529 (10.4)^b^	4806 (6.6)^b^	2733 (3.8)^b^	737 (1.0)^b^
No	94 295	26 899 (28.5)	4945 (5.2)	8375 (8.9)	16 577 (17.6)	8910 (9.5)	6371 (6.8)	1628 (1.7)
Alcohol abuse								
Yes	8995	3777 (42.0)^b^	1056 (11.7)^b^	1182 (13.1)^b^	2498 (27.8)^b^	1710 (19.0)^b^	910 (10.1)^b^	354 (3.9)^b^
No	157 687	36 727 (23.3)	6407 (4.1)	11 895 (7.5)	21 608 (13.7)	12 006 (7.6)	8194 (5.2)	2011 (1.3)
Substance abuse								
Yes	12 163	6035 (49.6)^b^	1718 (14.1)^b^	1864 (15.3)^b^	4192 (34.5)^b^	2851 (23.4)^b^	1480 (12.2)^b^	622 (5.1)^b^
No	154 519	34 469 (22.3)	5745 (3.7)	11 213 (7.3)	19 914 (12.9)	10 865 (7.0)	7624 (4.9)	1743 (1.1)

^a^ Percentages of HRSNs are derived from the numbers in this column.

^b^ *P* < .001.

^c^ *P* = .004.

^d^ *P* = .03.

^e^ *P* = .01.

In bivariate analysis, the proportions of patients utilizing IP or ED care during the 6 months prior to screening were significantly higher among patients who had HRSNs identified at baseline compared with those without needs (Table 3). This pattern was consistent across all HRSNs and both care outcomes with the exception of housing quality, where IP admissions were similar among those with and without needs. Differences in utilization were particularly notable among individuals who experienced needs related to housing stability, transportation, or safety. Of patients reporting housing instability, 26.9% (2006 of 7463) had an ED visit in the 6 months prior to screening compared with 14.3% (22 695 of 159 219) of those without this need. A total of 25.6% of patients (3512 of 13 716) with transportation needs had an ED visit in the prior 6 months compared with 13.9% of patients (21 189 of 152 966) without this need. Patients reporting interpersonal safety needs had the highest prevalence of ED use in the last 6 months (27.7% [656 of 2365]) compared with 14.6% (24 045 of 164 317) of those without a safety need.

**Table 3.** Crude Prevalence of Health Care Utilization in the 6 Months Prior to Screening by Patient Health-Related Social Need (HRSN) Status at Baseline

Baseline HRSN	Patients screened, No.^a^	Inpatient admission		Emergency department visit	
		No. (%)	*P* value	No. (%)	*P* value
Any					
No	126 178	8055 (6.4)	<.001	16 237 (12.9)	<.001
Yes	40 504	3215 (7.9)		8464 (20.9)	
Housing stability					
No	159 219	9858 (6.6)	<.001	22 695 (14.3)	<.001
Yes	7463	768 (10.3)		2006 (26.9)	
Housing quality					
No	153 605	10 419 (6.8)	.23	22 236 (14.5)	<.001
Yes	13 077	851 (6.5)		2465 (18.9)	
Food					
No	142 576	9333 (6.6)	<.001	19 219 (13.5)	<.001
Yes	24 106	1937 (8.0)		5482 (22.7)	
Transportation					
No	152 966	9961(6.5)	<.001	21 189 (13.9)	<.001
Yes	13 716	1309 (9.5)		3512 (25.6)	
Utilities					
No	157 578	10 530 (6.7)	<.001	22 615 (14.4)	<.001
Yes	9104	740 (8.1)		2086 (22.9)	
Interpersonal safety					
No	164 317	11 070 (6.7)	.001	24 045 (14.6)	<.001
Yes	2365	200 (8.5)		656 (27.7)	

^a^ Percentages of inpatient admissions and emergency department visits are derived from the numbers in this column.

**Figure 1.**
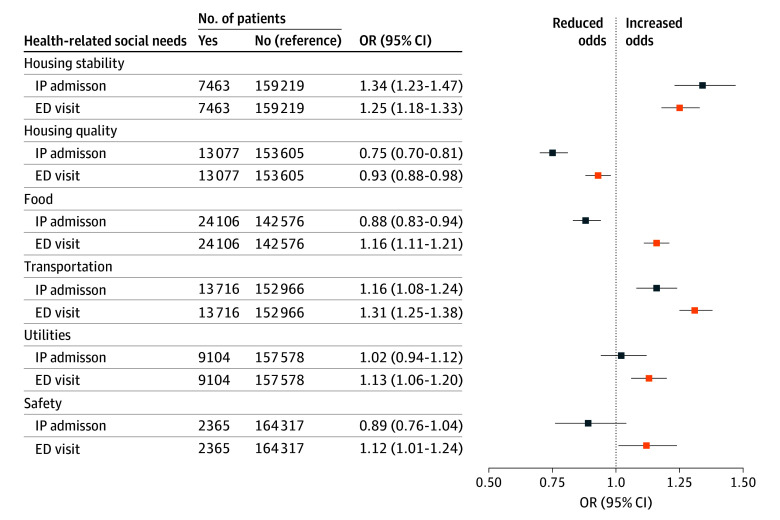
Adjusted Odds Ratios (ORs) of Inpatient (IP) Admission and Emergency Department (ED) Visits in the 6 Months Prior to Baseline Screening for Patients Screened (N = 166 682)

After adjustment for other HRSNs, demographic characteristics, and health conditions, only 2 HRSNs retained an association with an increase in IP utilization: housing stability (OR, 1.34; 95% CI, 1.23-1.47) and transportation (OR, 1.16; 95% CI, 1.08-1.24) (Figure 1). Conversely, patients with housing quality (OR, 0.75; 95% CI, 0.70-0.81) or food (OR, 0.88; 95% CI, 0.83-0.94) needs had significantly lower odds of IP admission in the 6 months prior to baseline HRSN screening. In models estimating ED use, all HRSNs were associated with utilization, with 5 HRSNs showing positive associations and 1 HRSN (housing quality) showing a negative association (Figure 1). The highest odds of ED use in final adjusted models were observed for transportation (OR, 1.31; 95% CI, 1.25-1.38) and housing stability (OR, 1.25; 95% CI, 1.18-1.33). Adjustment for health conditions had a larger association with the final estimates than adjustment for demographic characteristics (eTable 1 in Supplement 1).

There were 15 139 patients included in the longitudinal analysis of HRSN resolution and utilization (eTable 2 in Supplement 1). The mean (SD) time between baseline and follow-up screening was 13.6 (7.7) months. In bivariate analysis of this subsample, most (8650 of 15 139 [57.1%]) had fewer needs at the follow-up screening, 4052 of 15 139 (26.8%) had the same number of needs, and 2437 of 15 139 (16.1%) had more needs. Resolution ranged by HRSN: interpersonal safety (611 of 848 [72.1%]), utilities (2262 of 3359 [67.3%]), housing stability (1526 of 2365 [64.5%]), housing quality (2944 of 4689 [62.8%]), transportation (3097 of 4939 [62.7%]), and food (4395 of 9061 [48.5%]).

**Figure 2.**
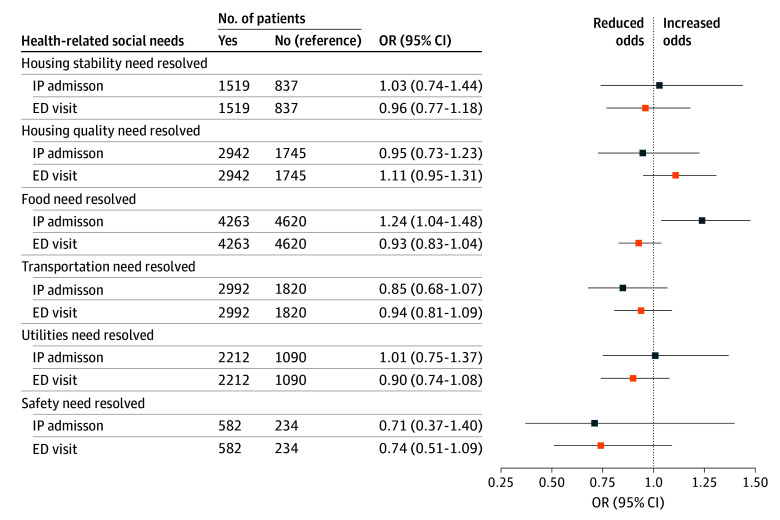
Adjusted Odds Ratios (ORs) of Inpatient (IP) Admission and Emergency Department (ED) Visits in the 6 Months After Follow-Up Screening for Patients With a Health-Related Social Need at Baseline and a Follow-Up Screening Available (N = 15 139) Numbers are based on the final adjusted models that excluded observations with missing data.

In bivariate analysis, we did not find any significant associations of resolution of HRSNs and subsequent IP admissions (eTable 3 in Supplement 1). However, resolution of 4 of the 6 HRSNs was associated with a decrease in ED visits (eTable 4 in Supplement 1). In multivariable models, resolution of individual HRSNs, after adjustment for other HRSNs present at follow-up, as well as demographic characteristics and comorbidities, was not associated with subsequent reductions in IP admission or ED visits (Figure 2). Resolution of 1 HRSN, food, was associated with an increase in IP admission (OR, 1.24; 95% CI 1.04-1.48). In contrast with baseline models, we did not see meaningful changes in estimates when adjusting sequentially for demographic characteristics and comorbidities (eTable 3 and eTable 4 in Supplement 1).

## Discussion

In this sample of Medicaid and Medicare beneficiaries seen at a large health system, nearly one-fourth (24.3%) had at least 1 HRSN when initially screened, with food and transportation needs being the most prevalent HRSNs. At baseline, only 2 needs (housing and transportation) were significantly and positively associated with IP admission in multivariable models, while 2 needs (housing quality and food insecurity) were negatively associated with IP admissions. Most HRSNs were positively associated with ED visits, except for housing quality. In the aim 2 analysis, HRSNs showed high levels of resolution between baseline and follow-up ranging from 48.5% (food) to 72.1% (interpersonal safety). After adjusting for other HRSNs present at follow-up, demographic characteristics, and comorbidities, resolution of baseline HRSNs was not associated with outcomes.

Prior studies have explored the association between HRSNs and health care utilization, but comparability with our findings is limited due to differences in study populations and adjustment for confounders. Here, we focus on findings reported from sites that implemented the AHC model or used the AHC screening tool. In their study of the AHC model in Houston, Holcomb et al^19^ operationalized HRSNs in terms of number (not type), used self-reported measures of ED visits, and did not adjust for comorbidities. In their study, odds of ED visits increased along with the number of HRSNs (housing, food, transportation, and/or utilities).

Other studies have been specific to Medicare Advantage beneficiaries. In 1 study, patients aged 65 years or older with at least 1 HRSN had 53.3% higher rates of avoidable hospitalizations along with higher rates of ED visits.^20^ Individual HRSNs except utilities were positively associated with ED visits and transportation had the strongest association with IP admissions and ED visits. This survey did not include interpersonal safety. In a study on Medicare Advantage beneficiaries with type 2 diabetes, lack of transportation and food insecurity were associated with higher rates of ED utilization and acute care hospitalization.^13^ Both of these studies adjusted for demographic characteristics as well as underlying health conditions using the Elixhauser Comorbidity Index.^30^ Our findings underscore the importance of accounting for comorbidities in analyses of HRSNs and health care utilization. However, the validity of the Elixhauser Comorbidity Index has been established only for hospitalization outcomes such as mortality.^30^ Summary scores also mask the variability between health conditions in their associations with HRSNs.^25^

Our study is novel in its examination of health service use after resolution of HRSNs. We observed significant crude associations for resolution of needs and ED visits. However, these associations were attenuated in multivariate models adjusting for other HRSNs present at follow-up followed by demographic characteristics and comorbidities. We saw the largest change in ORs in moving from crude to a model with other HRSNs at follow-up, suggesting strong associations among HRSNs. We found no other studies that examined resolution of HRSNs and subsequent health care utilization either within the AHC network or similar models. However, intensive interventions targeting specific needs have resulted in reduced care utilization. Examples include delivering food to nutritionally vulnerable individuals eligible for Medicaid or Medicare^5,31^ or provision of housing with supportive services for Medicare beneficiaries.^14^ In the context of the AHC model, the randomization of patients to either a resource list or navigation services did not result in a difference in resolution.^24^ This finding highlights the need to continue to strategize within health care settings about the best way to integrate services and partnerships to address HRSNs and to identify the best methods to address needs.

By better understanding how HRSNs are associated with health care utilization, health systems can focus their resources on targets that are most meaningful for their patient populations. Many payers are requesting screening procedures and HRSN needs data from provider organizations, and mechanisms exist through z-coding to allow for these data to be sent in a consistent manner to help reduce duplicative efforts to identify and support HRSNs. Creating mechanisms to document HRSNs and related codes in the EHR also support socially informed care from clinicians and care team members. Clinical settings have demonstrated successful integration of similar screening tools into the medical record, some with imbedded documentation of *ICD-10* codes, and the ability to generate resource referral guides for patients.^32,33^

### Strengths and Limitations

Our study has some strengths. One is our assessment of a large patient population within a single health system. This context provided the ability to screen a large and diverse sample of patients and to link HRSN data with valid measures of outcomes and covariates from the EHR. Adjusting for individual health conditions in the final models was key to elucidating the independent associations between HRSNs and care utilization. Another strength is our examination of discrete HRSNs rather than the counts of needs, both as independent variables and as covariates. The latter approach implies that all HRSNs have equal and additive associations with utilization, and that interventions need not target a specific need so long as they reduce the number of HRSNs experienced by patients. However, we found stronger associations with utilization for certain HRSNs, and more consistently for ED visits than for IP admissions. These findings suggest that HRSNs have unique relationships with health care outcomes and should be examined separately. Our study was unable to identify with precision the timing of resolution. Future studies or programmatic interventions may benefit from including procedures for follow-up with patients or referral agencies for communication on risk factor status to improve evaluation precision.

Our study also has some limitations. One is the use of measures intended for screening for a broad range of needs with varying degrees of resources. These measures were intended for referral for services rather than epidemiologic studies. For example, the housing quality measure used is very broad in asking about problems with pests such as bugs, ants or mice, mold, lead paint or pipes, lack of heat, water leaks, oven or stove not working, or smoke detectors missing. As this list covers a wide range of conditions, the measure likely captures a very heterogeneous population and may not be useful for measuring a level of need or examining associations with care needs. Similarly, the food measure may be broad in capturing responses about worry and experiences running out of food and thus people categorized as having this need may range widely in degrees of food insecurity. This wide range of need may explain some of the negative associations found in our study for these 2 HRSNs.

Another limitation is the lack of follow-up screening data for all patients with an identified HRSN at baseline. A comparison of those with needs at baseline who had follow-up screening vs those with no follow-up indicates the 2 groups are generally similar demographically, but the group with follow-up screenings has a higher prevalence of health conditions. Similarly, while we started with a large sample size, some models had smaller sample sizes (eg, interpersonal safety resolution) based on the number of patients with the need and multiple screening points. In addition, the study lacked a precise measure of timing of need resolution to guide examination of subsequent care utilization. Resolution may have happened any time between the first and second screening. In addition, while we were able to capture all visits in our health system, it is possible that patients utilized services outside of our system. Last, our measure of needs is based entirely on self-report, and disclosure of needs may be affected by many factors, including experiences with health care and social services organizations and may change over time.

## Conclusion

This cohort study found that individual HRSNs were uniquely associated with IP and ED utilization after adjusting for demographic characteristics and health conditions. The strongest associations were seen for ED utilization in cross-sectional analyses. Further research on the fluctuation of individual HRSNs over time is warranted to better understand if resolution of needs is associated with reduced health care utilization.

## Notes

Supplement 1. **eTable 1.** Estimates From Logistic Regression Models Predicting Inpatient Admission and Any ED Visit 6 Months Prior to Baseline HRSN Screening **eTable 2.** Among Patients With HRSNs at Baseline, Comparison of Those Included in the Resolution Analysis (Those With a Repeat Screening) and Those Excluded (Those Only 1 Screening) **eTable 3.** Estimates From Logistic Regression Models Predicting Inpatient Admission 6 Months After Follow-up HRSN Screening **eTable 4.** Estimates From Logistic Regression Models Predicting ED Visit 6 Months After Follow-up HRSN Screening

Supplement 2.
Data Sharing Statement
