# Supplementary material for: Health-Related Social Needs and Health Care Utilization in the Accountable Health Communities Model
Source: JAMA Netw Open. 2025 Dec 15;8(12):e2548036. doi: 10.1001/jamanetworkopen.2025.48036 (PMC12706677; doi:10.1001/jamanetworkopen.2025.48036)
Supplement: Supplement 1. — eTable 1. Estimates From Logistic Regression Models Predicting Inpatient Admission and Any ED Visit 6 Months Prior to Baseline HRSN Screening eTable 2. Among Patients With HRSNs at Baseline, Comparison of Those Included in the Resolution Analysis (Those With a Repeat Screening) and Those Excluded (Those Only 1 Screening) eTable 3. Estimates From Logistic Regression Models Predicting Inpatient Admission 6 Months After Follow-up HRSN Screening eTable 4. Estimates From Logistic Regression Models Predicting ED Visit 6 Months After Follow-up HRSN Screening [file jamanetwopen-e2548036-s001.pdf]

Supplementary Online Content

Sidebottom AC, Martins S, Vacquier MC, Dechaine C, Behrens D. Health-related social needs and health care utilization in the Accountable Health Communities model. *JAMA Netw Open.* 2025;8(12):e2548036. doi:10.1001/jamanetworkopen.2025.48036

eTable 1. Estimates From Logistic Regression Models Predicting Inpatient Admission and Any ED Visit 6 Months Prior to Baseline HRSN Screening
eTable 2. Among Patients With HRSNs at Baseline, Comparison of Those Included in the Resolution Analysis (Those With a Repeat Screening) and Those Excluded (Those Only 1 Screening)
eTable 3. Estimates From Logistic Regression Models Predicting Inpatient Admission 6 Months After Follow-up HRSN Screening
eTable 4. Estimates From Logistic Regression Models Predicting ED Visit 6 Months After Follow-up HRSN Screening

This supplementary material has been provided by the authors to give readers additional information about their work.

© 2025 Sidebottom AC et al. *JAMA Network Open.*

eTable 1. Estimates From Logistic Regression Models Predicting Inpatient Admission and Any ED Visit 6 Months Prior to Baseline HRSN Screening. Independent variables are presence of each HRSN at baseline (n = 166,682)

		Inpatient admission			ED visit		
		Model 1	Model 2	Model 3	Model 1	Model 2	Model 3
		OR (95% CI)	OR (95% CI)	OR (95% CI)	OR (95% CI)	OR (95% CI)	OR (95% CI)
Housing (stability)	No	ref.	ref.	ref.	ref.	ref.	ref.
	Yes	1.44 (1.32-1.57)	1.45 (1.33-1.58)	1.34 (1.23-1.47)	1.41 (1.33-1.50)	1.35 (1.27-1.43)	1.25 (1.18-1.33)
Housing (quality)	No	ref.	ref.	ref.	ref.	ref.	ref.
	Yes	0.82 (0.76-0.89)	0.82 (0.76-0.88)	0.75 (0.70-0.81)	1.00 (0.95-1.05)	1.00 (0.95-1.05)	0.93 (0.88-0.98)
Food	No	ref.	ref.	ref.	ref.	ref.	ref.
	Yes	1.06 (1.00-1.13)	1.04 (0.98-1.11)	0.88 (0.83-0.94)	1.44 (1.38-1.50)	1.33 (1.28-1.39)	1.16 (1.11-1.21)
Transportation	No	ref.	ref.	ref.	ref.	ref.	ref.
	Yes	1.39 (1.29-1.48)	1.37 (1.28-1.46)	1.16 (1.08-1.24)	1.58 (1.51-1.65)	1.50 (1.43-1.58)	1.31 (1.25-1.38)
Utilities	No	ref.	ref.	ref.	ref.	ref.	ref.
	Yes	1.06 (0.98-1.15)	1.08 (0.99-1.18)	1.02 (0.94-1.12)	1.18 (1.12-1.25)	1.17 (1.11-1.24)	1.13 (1.06-1.20)
Interpersonal safety	No	ref.	ref.	ref.	ref.	ref.	ref.
	Yes	0.97 (0.83-1.13)	0.96 (0.83-1.12)	0.89 (0.76-1.04)	1.28 (1.16-1.41)	1.23 (1.12-1.36)	1.12 (1.01-1.24)

Model 1: mutually adjusted for other baseline HRSNs.
Model 2: adjusted for other baseline HRSNs and demographics: sex, age, race/ethnicity, payor (Medicaid, Medicare, dual).
Model 3: adjusted for other baseline HRSNs, demographics, and comorbidities: asthma, type 2 diabetes, depression, hypertension, chronic kidney disease, chronic liver disease, chronic obstructive pulmonary disease, cardiovascular disease, alcohol/substance use disorder.

© 2025 Sidebottom AC et al. *JAMA Network Open.*

eTable 2. Among Patients With HRSNs at Baseline, Comparison of Those Included in the Resolution Analysis (Those With a Repeat Screening) and Those Excluded (Those Only 1 Screening)

	Unique patients with a need identified at baseline who were only screened once, or had 2nd screening within 6-months (n = 25,365) N (%)	Unique patients who had a need at baseline and who had a subsequent screening at least 6-months after (n = 15,139) N (%)
Sex		
Female	15,846 (62.5)	10,353 (68.4)
Male	9,519 (37.5)	4,786 (31.6)
Race/Ethnicity		
American Indian, NH	260 (1.0)	165 (1.1)
Asian/PI, NH	1,113 (4.4)	509 (3.4)
Black, NH	4,721 (18.6)	2,254 (14.9)
Hispanic/Latino	2,081 (8.2)	1,148 (7.6)
Multiracial, NH	700 (2.8)	418 (2.8)
White, NH	15,406 (60.7)	10,162 (67.1)
Missing	1,084 (4.3)	483 (3.2)
Age, mean(SD), years	37.01 (22.91)	41.82 (23.39)
0-17	5,788 (22.8)	2,777 (18.3)
18-44	10,584 (41.7)	5,370 (35.5)
45-64	5,052 (19.9)	3,742 (24.7)
65-84	3,608 (14.2)	3,067 (20.3)
85+	333 (1.3)	183 (1.2)
Payer		
Dual	1,368 (5.4)	1,194 (7.9)
Medicaid	19,092 (75.3)	9,861 (65.1)
Medicare	4,221 (16.6)	3,690 (24.4)
Other*	684 (2.7)	394 (2.6)

© 2025 Sidebottom AC et al. *JAMA Network Open.*

Language		
English	23,805 (93.9)	14,533 (96.0)
Somali	310 (1.2)	86 (0.6)
Spanish	518 (2.0)	244 (1.6)
Other	732 (2.9)	276 (1.8)
Health conditions at baseline		
Asthma	3,778 (14.9)	2,972 (19.6)
Diabetes (Type 2)	2,801 (11.0)	2,436 (16.1)
Depression	8,725 (34.4)	6,626 (43.8)
Hypertension	6,070 (23.9)	4,952 (32.7)
Chronic kidney disease	1,001 (4.0)	843 (5.6)
Chronic liver disease	8,14 (3.2)	622 (4.1)
COPD	1,361 (5.4)	1,182 (7.8)
Substance abuse	2,316 (9.1)	1,461 (9.7)
Alcohol abuse	3,669 (14.5)	2,366 (15.6)
ASCVD	1,865 (7.4)	1,502 (9.9)
CVD	7,563 (29.8)	6,042 (39.9)
BMI (adults only), mean(SD)	30.48 (8.14)	31.47 (8.51)
Number of screenings completed during time period		
1	25,258 (99.6)	0 (0)
2	107 (0.4)	9,094 (60.1)
3+	0 (0)	6,045 (39.9)

Abbreviations: NH — Non-Hispanic; SD — standard deviation; COPD — chronic obstructive pulmonary disease; ASCVD — atherosclerotic cardiovascular disease; CVD — cardiovascular disease; BMI — body mass index; GED — general educational development; k — 1,000.

© 2025 Sidebottom AC et al. *JAMA Network Open.*

eTable 3. Estimates From Logistic Regression Models Predicting Inpatient Admission 6 Months After Follow-up HRSN Screening. Independent variables are whether each need was resolved at follow-up among people with needs at baseline.

Note: Each table row represents a separate regression model.

	Crude	Model n	Model 1	Model 2	Model 3
	OR (95% CI)		OR (95% CI)	OR (95% CI)	OR (95% CI)
Housing (stability)		2,356			
Not Resolved	ref.		ref.	ref.	ref.
Resolved	1.05 (0.78-1.42)		1.07 (0.78-1.47)	1.10 (0.80-1.52)	1.03 (0.74-1.44)
Housing (quality)		4,687			
Not Resolved	ref.		ref.	ref.	ref.
Resolved	0.88 (0.69-1.12)		0.92 (0.72-1.18)	0.95 (0.74-1.21)	0.95 (0.73-1.23)
Food		8,883			
Not Resolved	ref.		ref.	ref.	ref.
Resolved	1.01 (0.87-1.18)		1.13 (0.95-1.34)	1.13 (0.95-1.35)	1.24 (1.04-1.48)
Transportation		4,812			
Not Resolved	ref.		ref.	ref.	ref.
Resolved	0.81 (0.67-0.99)		0.78 (0.62-0.97)	0.79 (0.63-0.99)	0.85 (0.68-1.07)
Utilities		3,302			
Not Resolved	ref.		ref.	ref.	ref.
Resolved	0.89 (0.69-1.15)		0.98 (0.73-1.30)	0.94 (0.70-1.26)	1.01 (0.75-1.37)
Interpersonal Safety		816			
Not Resolved	ref.		ref.	ref.	ref.
Resolved	0.89 (0.51-1.56)		0.81 (0.44-1.49)	0.78 (0.41-1.46)	0.71 (0.37-1.40)

Model 1: adjusted for other HRSNs present at follow-up screening.
Model 2 adjusted for other HRSNs present at follow-up screening and demographics: sex, age, race/ethnicity, payor (Medicaid, Medicare, dual).
Model 3: adjusted for other HRSNs present at follow-up screening, demographics, and comorbidities: asthma, type 2 diabetes, depression, hypertension, chronic kidney disease, chronic liver disease, chronic obstructive pulmonary disease, cardiovascular disease, alcohol/substance use disorder.

© 2025 Sidebottom AC et al. *JAMA Network Open.*

eTable 4. Estimates From Logistic Regression Models Predicting ED Visit 6 Months After Follow-up HRSN Screening. Independent variables are whether each need was resolved at follow-up among people with needs at baseline.

	Crude	Model n	Model 1	Model 2	Model 3
	OR (95% CI)		OR (95% CI)	OR (95% CI)	OR (95% CI)
Housing (stability)		2,356			
Not Resolved	ref.		ref.	ref.	ref.
Resolved	0.90 (0.75-1.10)		0.96 (0.78-1.18)	0.96 (0.78-1.18)	0.96 (0.77-1.18)
Housing (quality)		4,687			
Not Resolved	ref.		ref.	ref.	ref.
Resolved	0.94 (0.81-1.09)		1.07 (0.91-1.25)	1.10 (0.94-1.28)	1.11 (0.95-1.31)
Food		8,883			
Not Resolved	ref.		ref.	ref.	ref.
Resolved	0.75 (0.68-0.83)		0.85 (0.76-0.95)	0.87 (0.78-0.98)	0.93 (0.83-1.04)
Transportation		4,812			
Not Resolved	ref.		ref.	ref.	ref.
Resolved	0.78 (0.68-0.88)		0.88 (0.76-1.01)	0.89 (0.76-1.02)	0.94 (0.81-1.09)
Utilities		3,302			
Not Resolved	ref.		ref.	ref.	ref.
Resolved	0.69 (0.59-0.82)		0.86 (0.71-1.03)	0.87 (0.72-1.05)	0.90 (0.74-1.08)
Interpersonal Safety		816			
Not Resolved	ref.		ref.	ref.	ref.
Resolved	0.69 (0.50-0.96)		0.80 (0.56-1.14)	0.78 (0.54-1.12)	0.74 (0.51-1.09)

Note: Each table row represents a separate regression model.
Model 1: adjusted for other HRSNs present at follow-up screening.
Model 2 adjusted for other HRSNs present at follow-up screening and demographics: sex, age, race/ethnicity, payor (Medicaid, Medicare, dual).
Model 3: adjusted for other HRSNs present at follow-up screening, demographics, and comorbidities: asthma, type 2 diabetes, depression, hypertension, chronic kidney disease, chronic liver disease, chronic obstructive pulmonary disease, cardiovascular disease, alcohol/substance use disorder.

© 2025 Sidebottom AC et al. *JAMA Network Open.*
