# Supplementary material for: Health-Related Social Needs and Health Care Utilization in the Accountable Health Communities Model
Source: JAMA Netw Open. 2025 Dec 15;8(12):e2548036. doi: 10.1001/jamanetworkopen.2025.48036 (PMC12706677; doi:10.1001/jamanetworkopen.2025.48036)
Supplement: Supplement 2. — Data Sharing Statement [file jamanetwopen-e2548036-s002.pdf]

Data Sharing Statement

Sidebottom. Health-Related Social Needs and Health Care Utilization in the Accountable Health Communities Model. *JAMA Netw Open*. Published December 15, 2025. doi:10.1001/jamanetworkopen.2025.48036

Data
Data available: No

Additional Information
Explanation for why data not available: We would be willing to share data in the context of a data sharing agreement if interested parties reach out to the corresponding author.
